# A Cohort Clinical Study on the Efficacy of Topical Salicylic Acid/Piroctone Olamine Dandruff Pre‐Gel and Cleanser in Improving Symptoms of Moderate to Severe Seborrheic Dermatitis of the Scalp

**DOI:** 10.1111/jocd.16742

**Published:** 2025-01-07

**Authors:** Ling Ge, Zhiqing Liu, Shanhua Xu, Chuying Li, Meitong Jin, Yinli Luo, Yanli Kong, Jingbi Meng, Ge Zheng, Junzhi Gao, Ping Wang, Wei Bai, Heya Na, Xianchun Zhou, Zhehu Jin, Longquan Pi

**Affiliations:** ^1^ Department of Dermatology Yanbian University Hospital Jilin China; ^2^ Department of Medical Cosmetology Yanbian University Hospital Jilin China; ^3^ Yanbian University Medical College Jilin China; ^4^ Department of Anesthesia Yanbian University Hospital Jilin China; ^5^ Department of Internal Medicine Yanbian University Hospital Jilin China

**Keywords:** antimicrobial peptide, piroctone olamine, salicylic acid, seborrheic dermatitis, zinc PCA

## Abstract

**Background:**

Scalp seborrheic dermatitis (SD) is a chronic, recurrent inflammatory skin condition associated with scalp sebum secretion and dysbiosis.

**Aims:**

The aim of this study is to evaluate the efficacy and safety of a topical salicylic acid/piroctone olamine/zinc salt of L‐pyrrolidone carboxylate (Zinc PCA) scalp pre‐application gel in combination with a salicylic acid/piroctone olamine/antimicrobial peptide cleansing lotion for the treatment of moderate to severe scalp SD.

**Patients/Methods:**

In this prospective cohort study, 20 patients with moderate to severe scalp SD were treated with a combination of the scalp pre‐application gel and cleansing lotion for 4 weeks (one tube of the pre‐application gel per week and the cleansing lotion used every 1–3 days depending on the frequency of hair washing). This was followed by maintenance treatment with the cleansing lotion for 12 weeks. Efficacy was assessed through clinical and trichoscopic examinations, measuring the severity of dandruff, itching, erythema, and greasiness using a 4‐point scale.

**Results:**

This cohort study included a total of 20 patients with moderate to severe scalp SD. By the 4th week, the average dandruff score had significantly decreased from a baseline of 2.45 to 1.10 (*p* < 0.01). The average itchiness score reduced from 2.35 to 1.10 (*p* < 0.01), the erythema score dropped from 1.55 to 1.10 (*p* < 0.05), and the greasiness score decreased from 2.60 to 1.40 (*p* < 0.01). After 16 weeks, 12 patients with severe SD improved to mild SD, and six patients with moderate SD also improved to mild. During the treatment, two patients experienced a recurrence of dandruff, which subsequently resolved. The overall clinical improvement was 80%.

**Conclusions:**

Our study results indicate that the combination treatment of the scalp pre‐application gel and cleansing lotion is highly effective and safe for treating SD. The subsequent use of the cleansing lotion effectively maintains the benefits of the combined treatment. Trichoscopic examination provides accurate and reliable quantifiable data, aiding in the monitoring of treatment progress.

## 1 Introduction

Seborrheic dermatitis (SD) is a chronic, recurrent inflammatory disease characterized by clinical manifestations such as dandruff, erythema, and itching. It occurs in areas rich in sebaceous glands, particularly the scalp, face, and body folds. In adolescents and adults, it often presents as scalp dandruff. The occurrence of SD is attributed to various factors, including environmental factors (low temperature and humidity), microorganisms (*Malassezia*, *Staphylococcus*, etc.), impaired barrier function, immunosuppression, and neurogenic factors [1, 2, 3].

Currently, the primary treatment for scalp SD is topical therapy. Commonly used medications include selenium sulfide, ketoconazole, corticosteroids, and calcineurin inhibitors. Although these treatments can effectively alleviate symptoms in the short term, they have several notable drawbacks and potential side effects with long‐term use. Prolonged use of selenium sulfide may cause skin irritation, including redness, dryness, and even a burning sensation on the scalp, potentially leading to further damage to the skin barrier. A small number of patients may experience allergic reactions to ketoconazole, resulting in contact dermatitis. Long‐term use of corticosteroids can increase the risk of local infections, which can subsequently worsen the condition. Additionally, calcineurin inhibitors lack sufficient safety studies for extended use and may cause localized itching and burning sensations [4, 5, 6, 7]. Although these treatments are highly effective, long‐term use can lead to adverse reactions such as contact dermatitis and rashes, and there is a high recurrence rate [1, 8]. To avoid the long‐term use of medications, there is a need for a new safe and effective therapy with fewer side effects. Topical nondrug products may improve clinical outcomes. Lim et al. studied a shampoo containing piroctone olamine, climbazole, and caprylyl glycol as the main active ingredients. This shampoo effectively reduced the Adherent Scalp Flaking Score (ASFS), inhibited dandruff formation, and provided moisturizing and itch‐relieving effects [9]. The shampoo containing salicylic acid and 1% luliconazole resulted in improved quality of life for patients compared with treatment with ketoconazole shampoo. This enhanced the overall acceptance of treatment by patients [10]. The antibacterial and anti‐inflammatory effects of zinc PCA and antimicrobial peptides in SD have also been widely confirmed [11]. Nowadays, the treatment of scalp SD with non‐corticosteroid shampoos has become a research hotspot. However, the efficacy of using shampoo alone is limited. In this study, we aimed to investigate whether the clinical efficacy of combination therapy would be more significant by adding a pretreatment step to the existing shampoo regimen.

This study, conducted at our institution, included 20 patients with moderate to severe scalp SD. We observed the clinical efficacy and safety of a topical salicylic acid/piroctone olamine/zinc PCA scalp pre‐application gel in combination with a salicylic acid/piroctone olamine/antimicrobial peptide cleansing lotion for improving symptoms of scalp SD. The results are reported as follows.

## 2 Material and Methods

### 2.1 Study Design and Methodology

This study was approved by the ethics committee of Yanbian University Hospital (approval no. 2024031), and all participants provided informed consent. This clinical study was conducted in accordance with good clinical practice and the 1996 Helsinki Declaration. Inclusion criteria were ages 18 to 65, regardless of gender, with clinically diagnosed moderate to severe scalp SD. Exclusion criteria included the use of medications such as ketoconazole shampoo, glucocorticoids, spironolactone, finasteride, or other treatments that could affect scalp condition within the past 4 weeks; scalp conditions combined with psoriasis, tinea capitis, or other skin diseases; pregnancy or lactation; and any other reasons deemed unsuitable by the investigator.

All patients were treated with the scalp pre‐application gel (Medi‐hair Sixieqing Anti‐dandruff Cleaning Gel, Bohui Meicui Bioengineering Technology [Guangdong] Co. Ltd., China) in combination with the cleansing lotion (Medi‐hair Sixieqing Anti‐dandruff Refreshing Cleaning Cleanser, Bohui Meicui Bioengineering Technology [Guangdong] Co. Ltd., China) for 4 weeks. Prior to shampooing, the gel was evenly applied to the affected scalp area on dry hair, gently massaged for 5–10 min to allow sebum and dirt dissolution, and then rinsed off with water. The pre‐application gel was used once a week. The cleansing lotion was used every 1–3 days according to individual hair washing frequency. After lathering, the foam should be left on the affected area for 3–5 min before thoroughly rinsing. Follow‐up assessments were conducted to observe treatment efficacy. Patients continued to use the cleansing lotion for 12 weeks after the end of treatment, with follow‐up visits every 4 weeks to observe the maintenance effect of the cleansing lotion. To minimize potential confounding issues, all subjects were evaluated by a dermatologist. In accordance with the commercial confidentiality, the composition of the pre‐gel is outlined below. The only surfactant used for mild scalp pre‐cleansing is decyl glucoside. Salicylic acid, piroctone olamine, and PCA zinc are used for their antifungal and sebum‐regulating properties. Panthenol, glycerin, allantoin, hydrolysed corn starch, and bisabolol are moisturizing agents. In addition, the main ingredients of the cleansing lotion include sodium lauryl sulfate, sodium lauroyl sarcosinate, potassium coco‐glycinate, and coco‐methyl‐glucamine, which are used as surfactants in the formulation. These surfactants are commonly used for removing oils and dirt in shampoo formulations. The salicylic acid, piroctone olamine, and antimicrobial peptide are used as antifungal and antimicrobial agents. The antimicrobial peptide used in this study is a peptide antimicrobial substance produced by *Lactococcus lactis* . It is a lanolin sulfur bacteriocin composed of 34 amino acids. Other moisturizing agents are panthenol, glycerin, and niacinamide.

### 2.2 Subject Demographics

The demographic information of the study participants is as shown in Table 1.

**TABLE 1:** Demographics of patients (*n* = 20).

Content	
Gender, *n* (%)	
Male	8 (40)
Female	12 (60)
Age (years)	
Mean	27.05
Range	20–42
SD clinical severity (baseline), *n* (%)	
Moderate (5–8)	6 (30)
Severe (9–12)	14 (70)

### 2.3 Clinical Evaluation

The efficacy was assessed by measuring the severity of dandruff, itching, erythema, and greasiness at baseline, Week 1, Week 4, Week 8, Week 12, and Week 16. Evaluation was performed using a 4‐point scale based on ×20 trichoscopy (Dr. Camscope, Dermat Company, China) and macroscopic photographs (Canon, Japan). According to the ASFS scoring system, the scalp of each participant was divided into eight regions for assessment (left and right frontal regions, vertex, temporal regions, and occipital region). Each region was scored from 0 to 10 on the basis of the severity of scalp flaking: 0 = no flaking, 2 = slight flaking, 4 = some flaking, 6 = moderate flaking, 8 = severe flaking, and 10 = very severe flaking. The total ASFS score (ranging from 0 to 80) was then categorized into mild, moderate, and severe dandruff as follows: 1 = mild (few scattered flakes visible upon parting the hair, score 16–24), 2 = moderate (moderate amount of scattered flakes visible upon parting the hair, score 25–34), and 3 = severe (profuse flaking resembling snowflakes upon brushing or parting the hair, score 35–80). The assessment criteria for SD of the scalp were as follows: a total score of 1–4 indicates mild, 5–8 indicates moderate, and 9–12 indicates severe (refer to Table 2; [12, 13, 14, 15, 16, 17]). Clinical efficacy was determined using the Efficacy Index (EI) formula: (total score before treatment − Total score after treatment)/Total score before treatment × 100%. Marked improvement (EI ≥ 50%), moderate improvement (25% ≤ EI < 50%), and slight improvement (EI < 25%). “Clinical improvement” was considered when the score had improved by at least 50%, that is, marked improvement (EI ≥ 50%) [18].

**TABLE 2:** Clinical symptom score (4‐point scale).

Description	Rate
Clinical rating of dandruff	
No dandruff present	0
Mild: Few scattered flakes visible upon parting the hair	1
Moderate: Moderate amount of scattered flakes visible upon parting the hair	2
Severe: Profuse flaking of dandruff resembling snowflakes upon brushing or parting the hair	3
Clinical rating of erythema	
No erythema present	0
Mild: Few scattered pale red or yellow–red patches, erythema < 20% of scalp area, papules ≤ 5	1
Moderate: Multiple yellow–red patches with a few isolated papules, erythema covering 20% to 40% of the scalp area, 6 to 10 papules	2
Severe: Multiple infiltrated erythematous patches with numerous red papules, erythema covering ≥ 40% of the scalp area, > 10 papules	3
Clinical rating of greasiness	
No greasiness sensation on the second day after use	0
Mild: Slight greasiness sensation on the second day after use	1
Moderate: Moderate greasiness sensation on the second day after use	2
Severe: Significant greasiness sensation on the second day after use	3
Clinical rating of itching	
No itching sensation	0
Mild: Slight awareness of itching, no need to scratch, easily tolerable	1
Moderate: Noticeable itching sensation, occasional scratching, but tolerable and does not affect daily activities or sleep	2
Severe: Noticeable itching sensation, frequent scratching, unbearable, affects daily activities and sleep	3

### 2.4 Study Endpoints

The primary endpoints were to assess the combined treatment efficacy at Week 4 and the maintenance efficacy at Week 16.

### 2.5 Statistical Analysis

The data were analyzed using one‐way ANOVA test. Statistical analysis was conducted using GraphPad Prism with a significance level set at *p* < 0.05. Parametric data are presented as mean ± standard deviation.

## 3 Results

This cohort study included 20 patients with moderate to severe scalp SD, all of whom completed the treatment regimen. The primary endpoint evaluation took place at Week 4, followed by a maintenance phase extending through Week 16. The results showed that after 4 weeks of using a pre‐application scalp gel containing salicylic acid, piroctone olamine, and zinc PCA, in combination with a salicylic acid/piroctone olamine/antimicrobial peptide cleansing lotion, patients exhibited significant improvements in clinical symptoms (including dandruff, itching, erythema, and greasiness).

**FIGURE 1:**
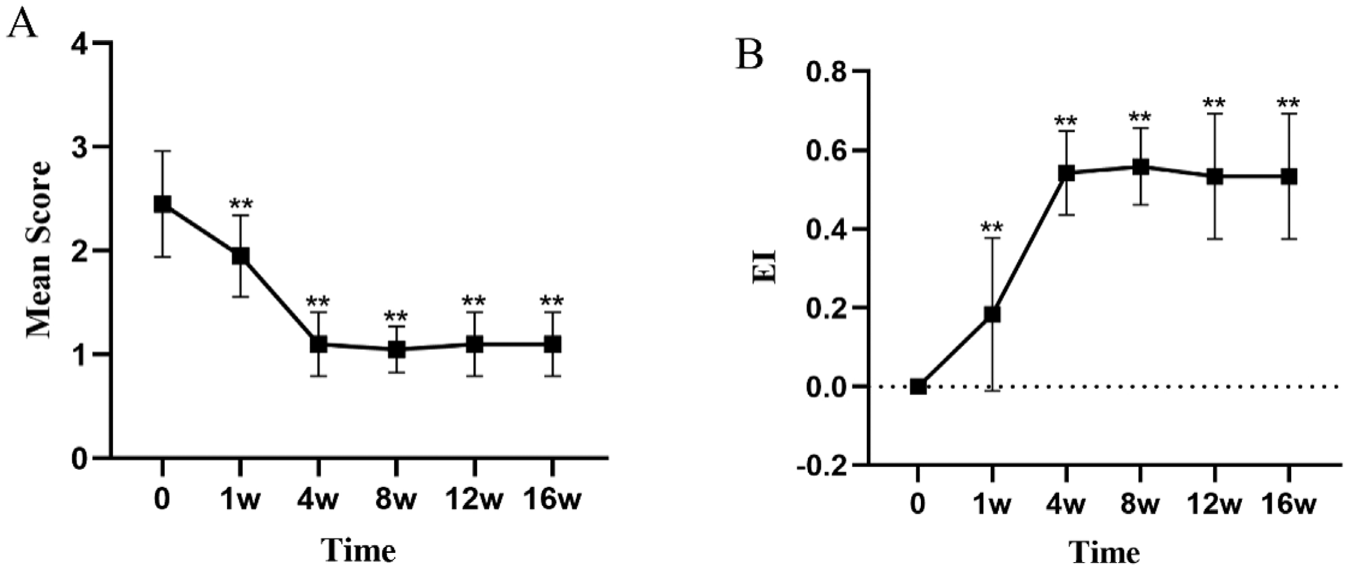
The trends in dandruff symptom scores (A) and the EI (B) over time during the treatment period are shown. Values at each time point are presented as means, and the error bars represent the standard deviation. **p < 0.01.

**FIGURE 2:**
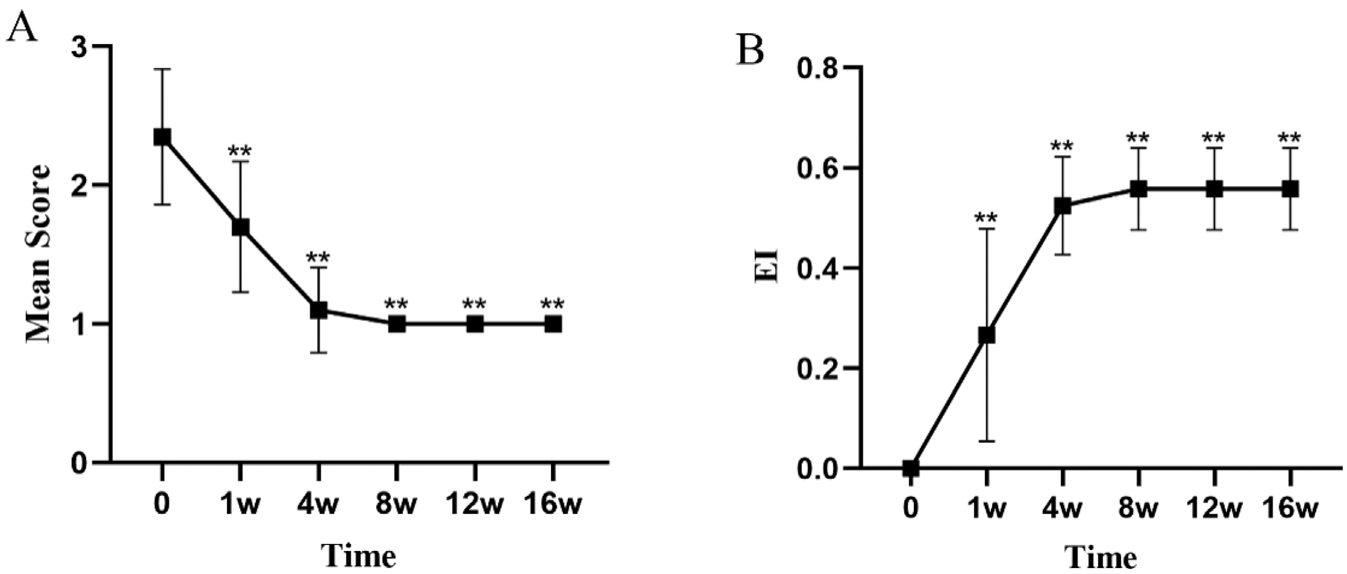
The trends in itching symptom scores (A) and the EI (B) over time during the treatment period are shown. Values at each time point are presented as means, and the error bars represent the standard deviation. **p < 0.01.

**FIGURE 3:**
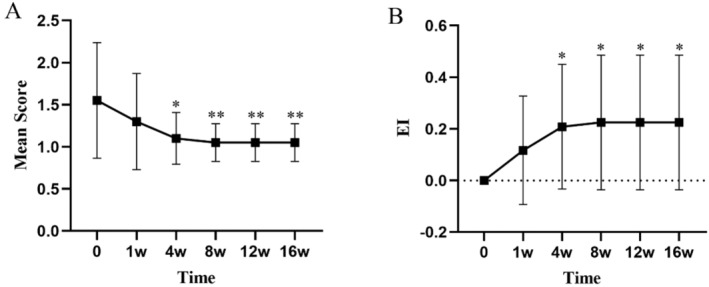
The trends in erythema symptom scores (A) and the EI (B) over time during the treatment period are shown. Values at each time point are presented as means, with error bars representing the standard deviation. *p < 0.05. **p < 0.01.

**FIGURE 4:**
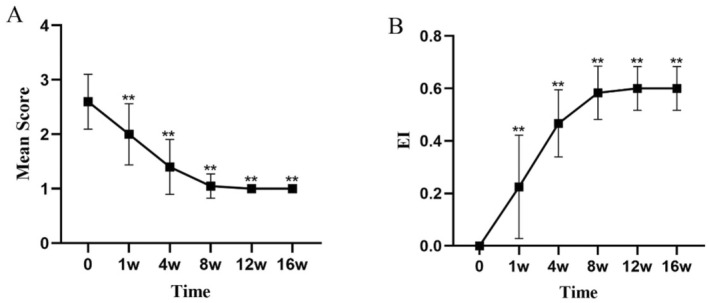
The trends in greasiness symptom scores (A) and the EI (B) over time during the treatment period are shown. Values at each time point are presented as means, with error bars representing the standard deviation. **p < 0.01.

**FIGURE 5:**
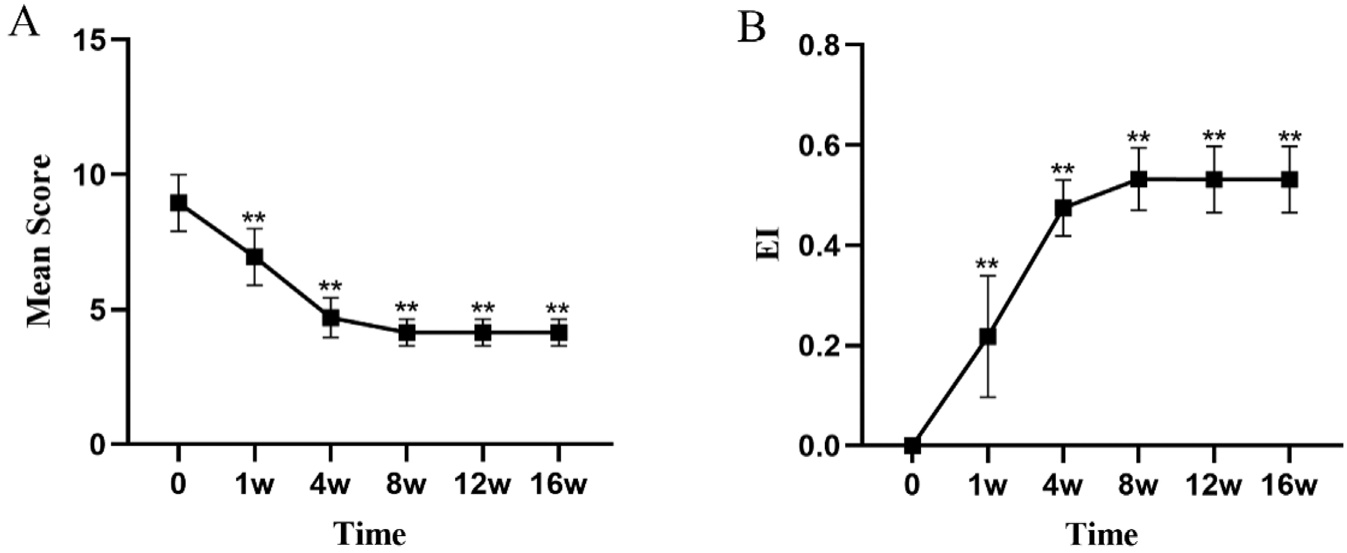
The trends in the total symptom scores (A) and the EI (B) over time during the treatment period are shown. Values at each time point are presented as means, with error bars representing the standard deviation. **p < 0.01.

All values are expressed as mean ± standard deviation (mean ± SD). At Week 4, the dandruff score significantly decreased from a baseline of 2.45 ± 0.5 to 1.10 ± 0.3 (*p* < 0.01), indicating a marked reduction in the severity of dandruff (Figure 1, Table 3). The itching score decreased from a baseline of 2.35 ± 0.48 to 1.00 ± 0.0 (*p* < 0.01), showing a significant alleviation of symptoms (Figure 2, Table 3). The erythema score decreased from a baseline of 1.55 ± 0.67 to 1.05 ± 0.22 at Week 4 (*p* < 0.05), with further improvement observed at subsequent time points (Figure 3, Table 3). The greasiness score decreased from a baseline of 2.60 ± 0.49 to 1.40 ± 0.49 at Week 4 (*p* < 0.01) and stabilized at 1.00 ± 0.0 from Week 8 onwards (Figure 4, Table 3). The overall score decreased from a baseline of 8.95 ± 1.05 to 4.7 ± 0.73 at Week 4 (*p* < 0.01) (Figure 5).

**TABLE 3:** Baseline and posttreatment symptom scoring (mean score).

Symptoms/Visits	Baseline	1 week	4 weeks	8 weeks	12 weeks	16 weeks
Dandruff	2.45	1.95 (*p* < 0.01)	1.10 (*p* < 0.01)	1.05 (*p* < 0.01)	1.10 (*p* < 0.01)	1.10 (*p* < 0.01)
Itching	2.35	1.70 (*p* < 0.01)	1.10 (*p* < 0.01)	1.00 (*p* < 0.01)	1.00 (*p* < 0.01)	1.00 (*p* < 0.01)
Erythema	1.55	1.30 (n.s.)	1.10 (*p* < 0.05)	1.05 (*p* < 0.01)	1.05 (*p* < 0.01)	1.05 (*p* < 0.01)
Greasiness	2.60	2.00 (*p* < 0.01)	1.40 (*p* < 0.01)	1.05 (*p* < 0.01)	1.00 (*p* < 0.01)	1.00 (*p* < 0.01)

At Week 16, the severity of SD was significantly reduced: six patients with moderate SD improved to mild SD, 12 patients with severe SD improved to mild SD, and 2 severe cases improved to moderate. Dandruff: After 16 weeks of treatment, eight patients with severe dandruff improved to mild, one severe case improved to moderate, and nine moderate cases improved to mild. Additionally, two moderate cases experienced symptom worsening during the follow‐up from Week 12 to 16. Itching: All patients experienced symptom improvement, with seven severe cases improving to mild and 13 moderate cases improving to mild. Erythema: The improvement rate for erythema was lower, with two severe cases improving to moderate and mild, respectively, and seven moderate cases improving to mild. Another 11 patients had mild erythema at baseline, with no significant worsening observed. Greasiness: Greasiness showed significant improvement, with 12 severe cases improving to mild and eight moderate cases improving to mild.

For clinical improvement, the treatment success rate for dandruff patients reached 90%. All patients with itching and greasiness demonstrated improvement in treatment outcomes, with no reports of recurrence. Erythema improved in 40% of patients (Table 4). Given that most patients enrolled had mild erythema, this may have influenced the statistical significance of the treatment effects on erythema. The overall treatment was well‐tolerated, with no adverse events reported. The overall clinical improvement reached 80% at Week 16 (Table 4). Between weeks 12 and 16, only two patients experienced a recurrence of dandruff, which was managed by increasing the frequency of the cleansing lotion.

**TABLE 4:** Clinical improvement during treatment, *n* (%).

	Dandruff	Itching	Erythema	Greasiness	Overall
4 weeks					
Marked improvement	18 (90)	18 (90)	7 (35)	12 (60)	10 (50)
Moderate improvement	2 (10)	2 (10)	2 (10)	8 (40)	10 (50)
Slight improvement	0	0	11 (55)	0	0
16 weeks					
Marked improvement	18 (90)	20 (100)	8 (40)	20 (100)	16 (80)
Moderate improvement	1 (5)	0	1 (5)	0	4 (20)
Slight improvement	1 (5)	0	11 (55)	0	0

**FIGURE 6:**
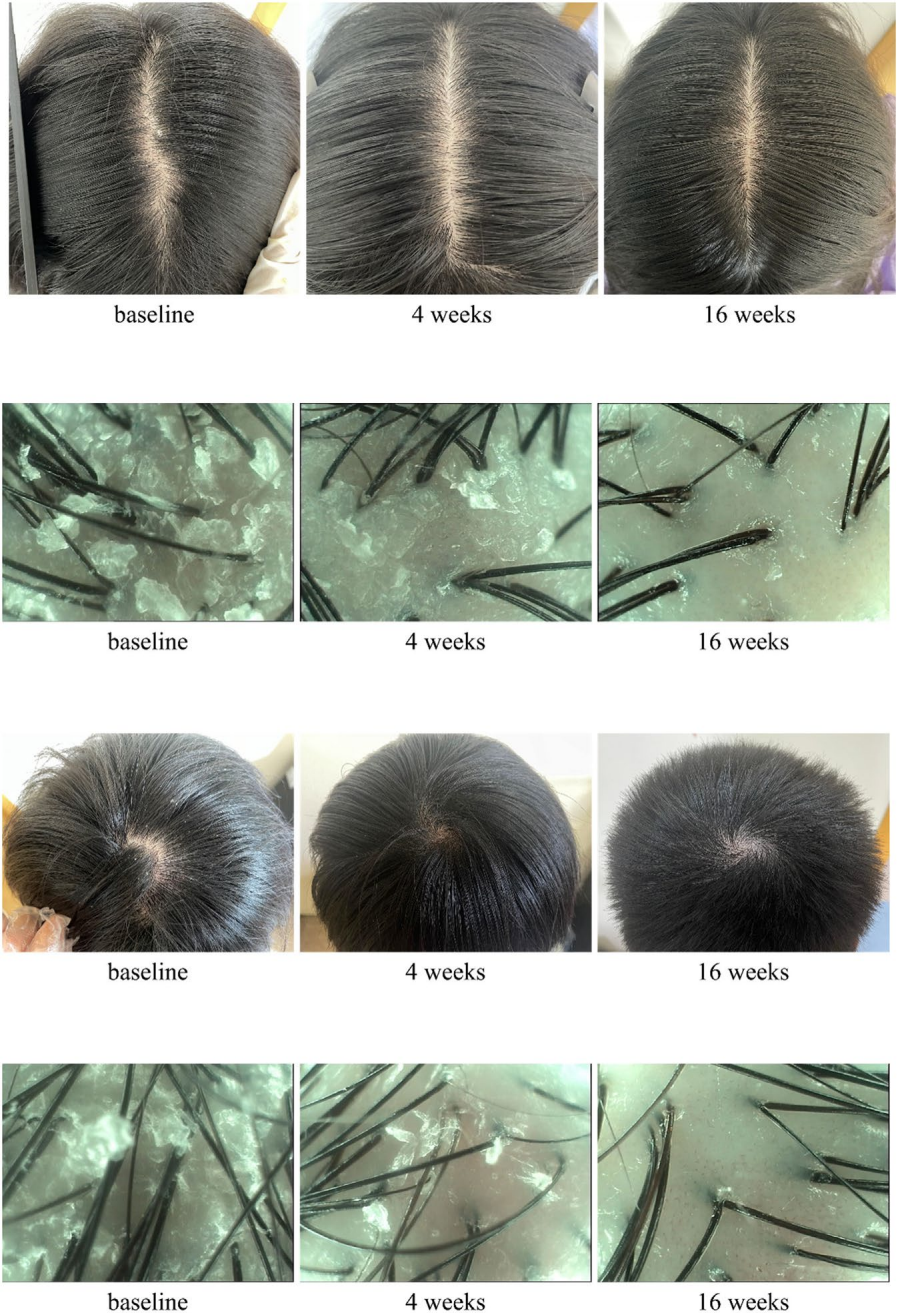
After 4 weeks of combination treatment, significant improvement in SD symptoms was observed, and continued improvement was observed during the subsequent 12 weeks of using the cleansing lotion.

In summary, the combined treatment regimen significantly improved symptoms of scalp SD by Week 4, and these improvements were maintained through Week 16 with continued use of the cleansing lotion, resulting in sustained and stable effects (Figure 6).

## 4 Discussion

SD is a common skin condition worldwide, with a prevalence of 1%–3% in the general population and a higher prevalence of 34%–83% in immunocompromised individuals. It exhibits a bimodal distribution, peaking in infants aged 2–12 months, during adolescence, and in early adulthood [19]. Previously, the most common treatments for SD were topical antifungal and anti‐inflammatory medications (such as steroids, calcineurin inhibitors, and lithium salts) [20]. Other widely used therapies include salicylic acid, benzoyl peroxide, sodium sulfacetamide, selenium sulfide, phototherapy, etc. Alternative therapies have also been reported, such as chamomile, pomegranate, tea tree oil, and glycyrrhizic acid complexes, all of which have shown potential in the treatment of SD [21, 22, 23]. To avoid adverse reactions associated with prolonged drug use, we investigated some non‐pharmaceutical ingredients. Piroctone olamine can penetrate cell membranes, bind to iron ions to form complexes, inhibit mitochondrial energy metabolism, and also inhibit the growth of *Malassezia*. When used alone or in combination with other drugs, piroctone olamine exhibits antifungal properties and can reduce the formation of dandruff [11, 20, 24, 25]. Salicylic acid is lipophilic, allowing it to penetrate the hair follicles and sebaceous glands, where it acts to dissolve keratin plugs and has anti‐inflammatory effects [21, 26]. Zinc PCA possesses antibacterial, anti‐inflammatory, and sebum‐regulating properties, synergizing with piroctone olamine. Zinc ions exert antibacterial effects by inhibiting bacterial fatty acid synthesis, disrupting their metabolic pathways, and damaging their cell membrane structure. It can also reduce skin inflammation by inhibiting the release of inflammatory mediators such as TNF‐α and IL‐1β. PCA is a component of the skin's natural moisturizing factor, which helps regulate sebaceous gland secretion [11, 27, 28]. Antimicrobial peptides are a class of small molecules that can kill or inhibit microorganisms such as bacteria, fungi, and viruses and are widely present in nature. They act by disrupting microbial membranes [29].

In this study, we introduced scalp pre‐application treatment in addition to the existing shampoo regimen. The gel contains piroctone olamine, salicylic acid, and zinc PCA, with higher concentrations of active ingredients compared with the cleansing lotion, providing more targeted therapy. Our research has demonstrated that adding pre‐application treatment once a week before shampooing significantly improves the efficacy of scalp SD treatment and enhances patient compliance.

There are several limitations in this study. Firstly, it employed a prospective cohort design without randomization, which may introduce selection bias and affect the representativeness and generalisability of the findings. Secondly, the sample size was relatively small and geographically concentrated, with only 20 patients, which may result in insufficient statistical power, particularly when significant individual variability is present, potentially impacting the accuracy of the results. Additionally, the follow‐up period in this study was 16 weeks, which may be insufficient to assess long‐term efficacy and relapse rates. Finally, the baseline erythema symptoms in the study population were generally mild, which may have limited the significance of the treatment effect and its applicability to other patient groups. Future research should involve larger, multicentre, and multiethnic randomized controlled trials, exploring responses in patients with varying baseline severity.

## 5 Conclusion

The combined therapy in this study utilized a two‐step treatment approach with a pre‐application gel and a cleansing lotion: the pre‐application gel, used before shampooing, delivers a high concentration of active ingredients, softens scales, reduces inflammation, and controls sebum production; the cleansing lotion, applied during shampooing, further removes loosened scales and excess oils, thereby reinforcing the therapeutic effects. Clinical practice has shown that this dual mechanism not only provides rapid symptom relief but also demonstrates a lower recurrence rate during the maintenance phase, suggesting advantages in long‐term management. From a practical perspective, this combined therapy is straightforward and user‐friendly, resulting in good patient compliance. The results of this study indicate that this combination therapy has significant clinical relevance and broad application potential in the management of SD, with the potential to become a standardized treatment for this condition.

## Author Contributions

Ling Ge designed the study, analyzed the data, and wrote the manuscript. Zhiqing Liu also contributed by writing the manuscript. Shanhua Xu and Chuying Li were involved in the investigation and data collection. Meitong Jin handled data curation. Data collection was further conducted by Yinli Luo, Yanli Kong, Jingbi Meng, Ge Zheng, Junzhi Gao, Ping Wang, Wei Bai, and Heya Na. Xianchun Zhou and Zhehu Jin contributed to the conceptualization and data curation. Longquan Pi was responsible for conceptualization and reviewed and revised the manuscript. All authors have read and approved the final manuscript.

## Ethics Statement

This study was approved by the ethics committee of Yanbian University Hospital (approval no.2024031). We certify that the study was performed in accordance with the 1964 Declaration of Helsinki and later amendments.

## Conflicts of Interest

The authors declare no conflicts of interest.

## Data Availability

The data that support the findings of this study are available from the corresponding authors upon reasonable request.
